# Crystal Structure of *Saccharomyces cerevisiae* ECM4, a Xi-Class Glutathione Transferase that Reacts with Glutathionyl-(hydro)quinones

**DOI:** 10.1371/journal.pone.0164678

**Published:** 2016-10-13

**Authors:** Mathieu Schwartz, Claude Didierjean, Arnaud Hecker, Jean-Michel Girardet, Mélanie Morel-Rouhier, Eric Gelhaye, Frédérique Favier

**Affiliations:** 1 Université de Lorraine, CRM2, UMR 7036, F-54500 Vandœuvre-lès-Nancy, France; 2 CNRS, CRM2, UMR 7036, F-54500 Vandœuvre-lès-Nancy, France; 3 Université de Lorraine, Interactions Arbres–Microorganismes, UMR1136, F-54500 Vandœuvre-lès-Nancy, France; 4 INRA, Interactions Arbres–Microorganismes, UMR1136, F-54280 Champenoux, France; Russian Academy of Medical Sciences, RUSSIAN FEDERATION

## Abstract

Glutathionyl-hydroquinone reductases (GHRs) belong to the recently characterized Xi-class of glutathione transferases (GSTXs) according to unique structural properties and are present in all but animal kingdoms. The GHR ScECM4 from the yeast *Saccharomyces cerevisiae* has been studied since 1997 when it was found to be potentially involved in cell-wall biosynthesis. Up to now and in spite of biological studies made on this enzyme, its physiological role remains challenging. The work here reports its crystallographic study. In addition to exhibiting the general GSTX structural features, ScECM4 shows extensions including a huge loop which contributes to the quaternary assembly. These structural extensions are probably specific to *Saccharomycetaceae*. Soaking of ScECM4 crystals with GS-menadione results in a structure where glutathione forms a mixed disulfide bond with the cysteine 46. Solution studies confirm that ScECM4 has reductase activity for GS-menadione in presence of glutathione. Moreover, the high resolution structures allowed us to propose new roles of conserved residues of the active site to assist the cysteine 46 during the catalytic act.

## Introduction

Glutathionyl-hydroquinone reductases (GHRs) belong to the glutathione transferase (GST) family of enzymes and catalyze efficiently the reduction of hydrophobic, bulky glutathionyl-hydroquinones [1]. These proteins are clustered within the structural Xi-class [2] well established in bacteria, archaea, plants, protozoa and fungi including the yeast *Saccharomyces cerevisiae* [3–5]. Although the physiological role of the bacterial GHR PcpF from *Sphingobium chlorophenolicum* (ScPcpF) is well established in the pentachlorophenol degradation pathway [6], the role of these enzymes in other prokaryotes and in eukaryotes is still challenging. They could have a function in biochemical pathways that involve quinone reduction [1].

One of the most studied GHRs from a biochemical point of view is the protein ECM4 from the yeast *Saccharomyces cerevisiae* (ScECM4). This enzyme was characterized as a thiol-transferase [7]. Site-directed mutagenesis experiments allowed the authors to highlight the residues important for the thiol-transferase activity of the enzyme including the catalytic cysteine 46. Later, it was demonstrated that this cytosolic enzyme could reduce various glutathionyl-hydroquinones (GS-hydroquinones) such as GS-hydroxy-p-hydroquinone, GS-methyl-p-hydroquinone, GS-hydroquinone and GS-menadiol [1]. Molecular biology studies suggested that this enzyme could be involved in cell wall biosynthesis [8]. Moreover, ECM4 gene expression was induced by genotoxic [9] and oxidative agents [10]. Likewise, a cytotoxicity study of menadione in *S*. *cerevisiae* pointed to ScECM4 as a GST that might be involved in cellular protection [11]. According to sequence similarity, ScECM4 was initially classified in the Omega-class GST and named Gto2 [7]. Later, according to phylogenetic studies and for its ability to reduce GS-hydroquinones, ECM4 was moved to the GHR family [12].

Resolution of the first GHR structure (*Phanerochaete chrysosporium* PcGHR1, pdb code 3PPU) revealed unique structural properties [2]. Its canonical GST fold showed a new dimerization mode by the helical C-terminal domain and a new active site including a triad of tyrosines. These features led to establish a new structural Xi class (GSTX) for these GHRs. Up to now, structures of GHRs from Poplar (PtGHR1) [13] and from the bacteria *Escherichia coli* (EcYqjG) and *Sphingobium chlorophenolicum* (ScPcpF) [14] have been described. In addition, the Xi-class also contained proteins for which GHR activity was not established (PDB entries 3M1G and 4PTS).

In this study, we described the X-ray structures of ScECM4 and glutathionylated ScECM4 (ScECM4-SG). These structures obtained at near atomic resolution constituted the first yeast GHR models. Comparison of ScECM4 with other GSTXs from the PDB revealed additional features, the most striking ones being a huge loop β2-α2, an extension of the helix α5 and the presence of an additional helix α5’. According to sequence alignment, these specificities seemed to be conserved in the genus *Saccharomyces* and closely related genera. Moreover, the structures allowed a fine analysis of the active site and provided complementary information for the comprehension of the catalytic mechanism of this enzyme. Activity measurements associated with structural results showed the reduction of GS-menadione to menadione catalyzed by ScECM4.

## Material and Methods

### Cloning in bacterial expression vector

The sequence encoding ScECM4 (YKR076W) was amplified by polymerase chain reaction (PCR) from *Saccharomyces cerevisiae* genomic DNA as template and 5’-GGGGGGCATATGTCGAAACAGTGGGCGAG and 5’-GGGGGGCTCGAGTAAAGGACGAATATCTGGCTTG as specific forward and reverse primers, respectively. Amplified sequence was subsequently digested and cloned into pET-26b plasmid (Novagen) between *Nde*I and *Xho*I restriction sites, allowing the fusion of a His-Tag at the C-terminal part of ScECM4.

### Expression and purification of recombinant ScECM4

The expression of recombinant ScECM4 was performed at 37°C using *E*. *coli* Rosetta2 (DE3) pLysS strain (Novagen) transformed with pET-26b::ScECM4 in LB medium supplemented with kanamycin (50 μg/mL) and chloramphenicol (34 μg/mL). When the cell culture reached an OD_600nm_ of 0.7, 0.5% (v/v) of ethanol was added to the culture medium and cells were cooled to 4°C for 3 h. Protein expression was then induced with 0.1 mM isopropyl β-D-1-thiogalactopyranoside (IPTG) and cells further grown for 18 h at 20°C. Cells were then harvested by centrifugation, resuspended in a 30 mM Tris-HCl buffer, pH 8.0, containing 200 mM NaCl and stored overnight at -20°C. Cell lysis was performed by sonication. The cell extract was centrifuged at 20,000 *g* for 25 min at 4°C to remove cellular debris and aggregated proteins. After the addition of 10 mM imidazole, ScECM4 was purified from the soluble extract by gravity-flow chromatography on a nickel nitrilotriacetate (Ni-NTA) agarose resin according to the manufacturer’s recommendations followed by a gel filtration step at 4°C on a Superdex^TM^200 16/600 column connected to an ÄKTA-Purifier^TM^ (GE Healthcare) and finally stored in a 30 mM Tris-HCl buffer, pH 8.0, containing 200 mM NaCl at -20°C. Concentration of ScECM4 recombinant protein was determined at 280 nm using a theoretical molar absorption coefficient of 84800 M^-1^.cm^-1^.

### Crystallization

Crystallization of ScECM4 was assayed by the microbatch under paraffin oil method at 278 K. ScECM4 (41 mg/mL) crystallized by mixing 1 μL of protein with 1 μL of commercial crystallization solution (Wizard I n°10 (Rigaku), consisting of 20% (w/v) polyethyleneglycol 2000 monomethylether in 0.1 M Tris-HCl buffer, pH 7.0).

ScECM4-SG crystals were equally obtained either by soaking technics (by adding 5 mM GS-menadione to the drop containing crystals of the apo enzyme) or by co-crystallization (by mixing 5 mM GS-menadione to the drop containing the protein and the precipitating agent before crystallization).

### Data collection, processing and refinement

ScECM4 and ScECM4-SG crystals were flash-frozen after a quick soaking in the mother liquor containing 20% (v/v) glycerol as a cryoprotectant. X-ray diffraction experiments were carried out at 100 K on the ESRF beamline FIP BM30A (Grenoble, France). ScECM4 and ScECM4-SG crystals diffracted respectively to 1.45 Å and 1.68 Å according to the CC_1/2_ correlation coefficient [15]. Data sets were indexed and integrated with XDS [16] and scaled with XSCALE [16]. The data sets were complete to 1.5 Å and 1.8 Å resolution for ScECM4 and ScECM4-SG, respectively. The structure of ScECM4 was solved by the molecular replacement method using MOLREP of the CCP4 package [17] with the coordinates of PcGHR1 (PDB code 3PPU) [2] as the search model. Structures were refined with PHENIX [18] and built with COOT [19]. After the final refinement/building cycle, atoms that were unobserved in the electron density notably for side chains of surface residues were set with a null occupation and with a B-factor fixed to the respective mean B-factor of the structure. Validation of both structures was performed using MolProbity [20] and the wwPDB Validation server [21, 22].

Coordinates and structure factors of ScECM4 and ScECM4-SG have been deposited in the Protein Data Bank under accession codes 5LKB and 5LKD, respectively. Their PDB Validation reports are provided as supplementary materials (S1 and S2 Files, respectively).

### Activity measurements

GS-menadione was prepared according to Lallement *et al*. [13]. Catalytic reduction of GS-menadione to menadione by ScECM4 was evaluated according to two previously described protocols [13]. In a first experiment, 1 μM ScECM4 was added to 500 μL of a 30 mM Tris-HCl buffer, pH 8.0, containing 1 mM EDTA, 100 μM GS-menadione and various concentrations of GSH (0.05 to 1 mM). Reduction of GS-menadione to menadione was followed by monitoring absorbance from 280 to 480 nm. GS-menadione exhibited a maximum of absorption at 430 nm that disappeared upon reduction within 10 minutes. A control performed without ScECM4 or without glutathione resulted in no change of absorbance.

In a second experiment, menadione released by the reaction was isolated by HPLC and detected spectrophotometrically. ScECM4 was added at 10 μM in a reaction volume of 600 μL of 30 mM Tris-HCl buffer, pH 8.0, containing 1 mM EDTA, 2 mM GSH and 1 mM GS-menadione. After 10 minutes of reaction at room temperature, 40% (v/v) ethanol was added to the mixture and the tubes were vigorously shaken during 30 s to stop the reaction. After centrifugation (10 min, 14 000 *g*), products were separated by HPLC onto a Gemini C18 column (150 x 3 mm internal diameter, 5-μM particle size, 10-nm porosity; Phenomenex) equilibrated with 30 mM acetic acid buffer, pH 4.16 containing 10% (v/v) acetonitrile. A stepwise gradient of acetonitrile in the acetic acid buffer was performed at flow-rate of 1 mL/min: 10–50% linear gradient for 5 min, then 50–100% linear gradient for 4 min, and finally isocratic elution at 10% (v/v) of acetonitrile for 9 min. Detection was monitored at 250 nm.

### Bioinformatics

For the structural alignment, atomic coordinates of five distinct Xi GSTs were selected (PDB codes 3PPU, 4USS, 4G0K, 4FQU, 4PTS). The structural alignment was performed with the module Multiseq [23] integrated in VMD [24] and manually annotated.

For the phylogenetic analysis of yeast ECM4-like proteins, the sequence of ScECM4 (accession number NP_013002) was used in BLASTP on the Joint Genome Institute (JGI) Website to query the fungal genome database [25], or in BLASTP on the National Center for Biotechnology Information (NCBI) website. Multiple sequence alignment was made using Promals3D [26] with the structure of ScECM4 as input. The sequence alignment was manually annotated to highlight the secondary structure element and the putative residues of the active site.

## Results and Discussion

### ScECM4 crystals soaked with GS-menadione results in a glutathionylated enzyme

Crystallization of ScECM4 occurred quickly in about 4 h. Although polycrystals were frequently observed, several monocrystals suitable for X-ray diffraction experiments have been obtained (Table 1). One of our purposes was to obtain a high resolution structure of the complex between ScECM4 and GS-menadione, which at the moment was thought to be an analog of the substrate GS-menadiol [1]. A previous study reported the complex obtained between the bacterial GHR EcYqjG and GS-menadione, however the electron density corresponding to the menadione moiety was found to be diffuse [14]. To achieve our goal, diffusion experiments were performed in ScECM4 crystals. After a one-day soaking with GS-menadione, crystals appeared unaltered and exhibited a yellowish color due to a probable absorption of GS-menadione. Crystals with the same aspect were obtained by the cocrystallisation method. Three X-ray diffraction data sets were collected (ScECM4, ScECM4 soaked with GS-menadione and ScECM4 cocrystallized with GS-menadione) and analyzed for each experiment.

**Table 1:** Diffraction and refinement statistics.

		ScECM4	ScECM4-SG
**Diffraction data**			
	Diffraction source	FIP-BM30A, ESRF	FIP-BM30A, ESRF
	Detector	ADSC Q315r CCD	ADSC Q315r CCD
	Wavelength (Å)	0.97977	0.97977
	Unit-cell parameters		
	a, b, c (Å)	55.51 82.96 80.65	55.44 82.44 80.58
	β (°)	95.1	95.3
	Space group	*P*2_1_	*P*2_1_
	Resolution range (Å)	46.01–1.45 (1.49–1.45)	47.59–1.68 (1.72–1.68)
	Total No. of reflections	442,863 (19591)	299,262 (16693)
	No. of unique reflections	124,109 (7335)	78,066 (4463)
	Average multiplicity	3.6 (2.7)	3.8 (3.7)
	Mean I/σ(I)	19.7 (1.9)	17.9 (1.9)
	Completeness (%)	96.4 (77.0)	95.0 (74.3)
	R_merge_ (%)	3.5 (51.1)	5.5 (70.6)
	R_meas_ (%)	4.2 (64.3)	6.4 (82.4)
	CC_1/2_ (%)	99.9 (76.1)	99.9 (75.8)
	Wilson B factor (Å^2^)	15.1	16.9
**Refinement**			
	Resolution range (Å)	46.01–1.45 (1.50–1.45)	47.59–1.68 (1.74–1.68)
	R_work_ (%)	16.7 (30.4)	15.9 (27.5)
	R_free_ (%)	19.4 (30.3)	20.1 (32.3)
	No. of protein atoms	11 305	11 284
	No. of waters	785	638
	Ligands	3 GOL	2 GSH
	Average B factor (Å^2^)	22.9	25.7
**Model quality**			
	RMSZ bonds lengths	0.76	0.9
	RMSZ bond angles	0.84	0.89
	RSRZ outliers (%)	1	0
	Ramachandran favored (%)	98.4	98.1
	Ramachandran outliers (%)	0	0
	Molprobity rotamer outliers (%)	0	0.5
	Molprobity clashscore	0.82	1.28
	Molprobity score	0.75	0.85
**PDB entry**		5LKB	5LKD

R_merge_ = ∑_*hkl*_∑_*i*_|*I*_*i*_(*hkl*) − ⟨*I*(*hkl*)⟩|/∑_*hkl*_∑_*i*_*I*_*i*_(*hkl*). R_meas_ = ∑_*hkl*_{*N*(*hkl*)/[*N*(*hkl*) − 1]}^1/2^ ∑_*i*_|*I*_*i*_(*hkl*) – ⟨*I*(*hkl*)⟩|/∑_*hkl*_∑_*i*_*I*_*i*_(*hkl*). CC_1/2_ is the correlation coefficient of the mean intensities between two random half-sets of data. R_work_ = ∑_*hkl*_||*F*_*obs*_| − |*F*_*calc*_||/∑_*hkl*_|*F*_*obs*_|. 5% of reflections were selected for R_free_ calculation. Ligands PDB codes GOL: glycerol, GSH: glutathione. R.m.s.z.: root mean square Z-score. R.s.r.z.: real space refinement Z-score (indicate the fit between residue and electron density derived from experimental data, according to the wwPDB validation server). The molprobity clashscore is the number of serious clashes per 1000 atoms. The molprobity score is a log-weighted combination of the clashscore, percentage Ramachandran not favored and percentage bad side-chain rotamers. Values in parentheses are for highest resolution shell.

**Fig 1:**
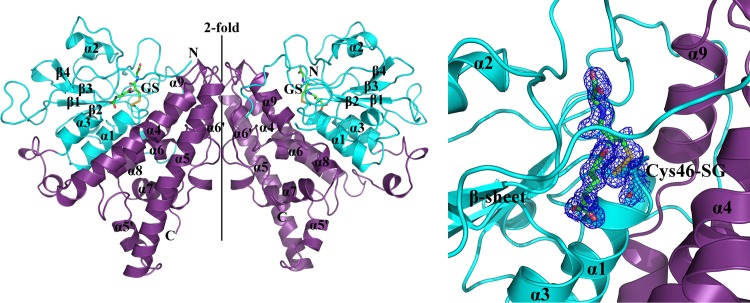
Crystal structure of glutathionylated ScECM4. **Left**: Cartoon representation of the dimer of glutathionylated ScECM4 (cyan: N-terminal domain, violet: C-terminal domain). The monomers are related by a two-fold axis symbolized as a solid line. Glutathione is represented as green sticks. **Right**: Electron density around the glutathione and cysteine 46 highlighting the disulfide bond. The 2mFo-DFc map contoured at 1.0 sigma is a composite omit map calculated by Phenix [18].

Structures of ScECM4, ScECM4 soaked with GS-menadione and ScECM4 cocrystallized with GS-menadione were solved at 1.45 Å, 1.68 Å and 1.72 Å, respectively (Fig 1). All crystals were isomorphous and belonged to space group *P*2_1_. The asymmetric unit contained two monomers related by a 2-fold axis. ScECM4 exhibited no GSH in the active site but rather glycerol molecules. Surprisingly, both soaked and cocrystallized forms with GS-menadione resulted in a clear electron density that was unambiguously interpreted as glutathione covalently bound to C46 of ScECM4 (ScECM4-SG), while no GS-menadione was detected in the electron density (Fig 1). Since no differences were found between both structures in complex with glutathione, we decided to use the one with the highest resolution, corresponding to the soaking experiment (Table 1). Due to absence or weakness of electron density in some regions, all monomers built in the structures lacked about thirty residues at similar positions, namely the ten first N-terminal ones (fifteen in monomer B of ScECM4-SG), the 6 histidine residues from the C-terminal tag and residues between D83 and L95. For the latter, a possible explanation was the high amount of charged residues in this region. Residues R106 to S115 were also absent with the exception of monomer A of ScECM4-SG where a poor electron density was observed, leading to high B-factors for these atoms with respect to the overall B-factor.

**Fig 2:**
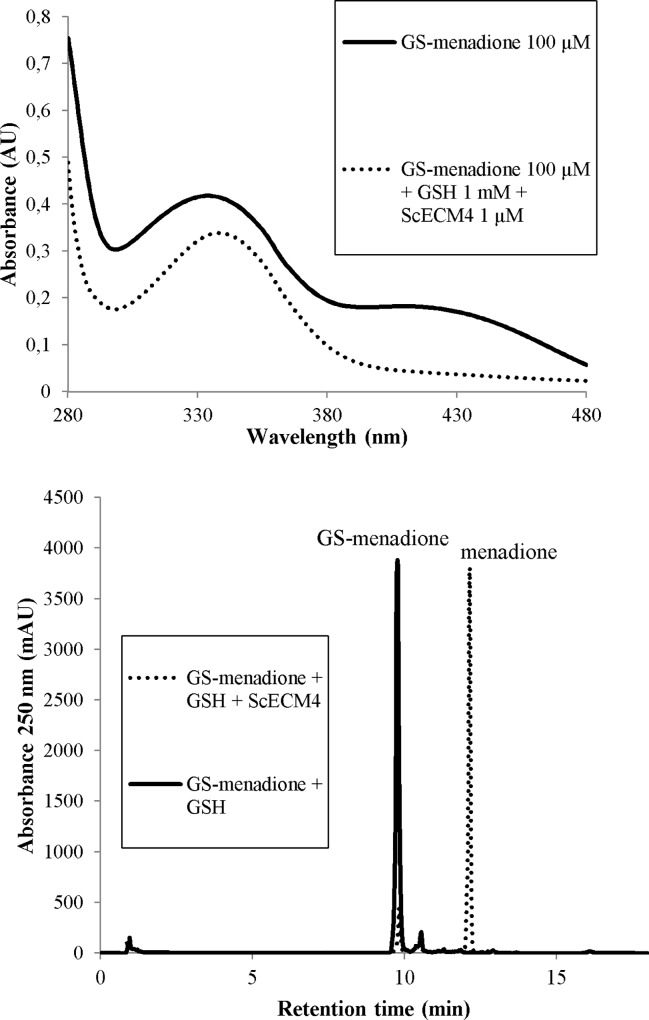
Activity of ScECM4 with GS-menadione. **Top,** UV/visible spectrum of 100 μM GS-menadione in Tris-EDTA buffer, as recorded before addition of 1 μM ScECM4 and 1 mM GSH (solid line), and then after 10 min of reaction with these compounds (dotted line). **Bottom,** HPLC profile at 250 nm after 15 min incubation of ScECM4 with GS-menadione and GSH. GS-menadione shows a typical peak at a retention time of 10 min (solid line) and menadione at 12 min (dotted line).

Since the soaking and the cocrystallization experiments led to a mixed disulfide bond between C46 and glutathione, we investigated whether ScECM4 could have broken up the GS-menadione bond. First, we eliminated the possibility of a direct reaction of ScECM4 with glutathione. Soaking experiments of crystals with reduced (GSH) or oxidized (GSSG) glutathione only did not result in a covalent complex (data not shown). Then, activity of ScECM4 with GS-menadione was assessed either by direct spectrophotometry measurement at 430 nm or by HPLC separation where menadione was eluted at retention time of 12 min [13] and GS-menadione, more hydrophilic, at 10 min (Fig 2). A mixture of 1 μM ScECM4 and 100 μM GS-menadione failed to consume GS-menadione. The addition of 1 mM GSH in the mixture allowed a continuous catalytic cycle and resulted in solution fading (*i*.*e*. disappearance of the yellowish color due to GS-menadione). Assays with decreasing concentrations of GSH (1 to 0.05 mM) revealed a concomitant decrease of activity (S1 Fig). The need for an excess of glutathione could be explained by the high *K*_m_ value of ScECM4 for GSH (350 μM previously determined with 600 μM of GS-methylhydroquinone [1]). Altogether, these ScECM4 activity measurements stated for reduction of GS-menadione to menadione in presence of glutathione. Earlier, Lam *et al*. [1] observed a good activity of ECM4 with the reduced form of GS-menadione (*i*.*e*. GS-menadiol). Thus, ScECM4 resembled YqjG from *E*. *coli*, GHR1 from poplar and GHR1 from *P*. *chrysosporium* in their ability to catalyze the reduction of glutathionyl-(hydro)quinones [13].

These results explained why enzyme glutathionylation was observed after soaking experiments of ScECM4 crystals with GS-menadione. The protein was likely active in the crystals grown at pH 7.0. On the contrary, Green and colleagues succeeded to obtain crystals of the complex between EcYqjG and GS-menadione [14]. Since EcYqjG displayed optimal activity with GS-menadione at pH 8.0 [13] while the cysteine pKa was estimated to be 7.3 [14], crystals of complexes were probably obtained thanks to crystallization conditions (pH 6.5, 1.6 M ammonium sulfate) which could have blocked catalysis.

### ScECM4 is a GST Xi with features specific to *Saccharomycetaceae*

**Fig 3:**
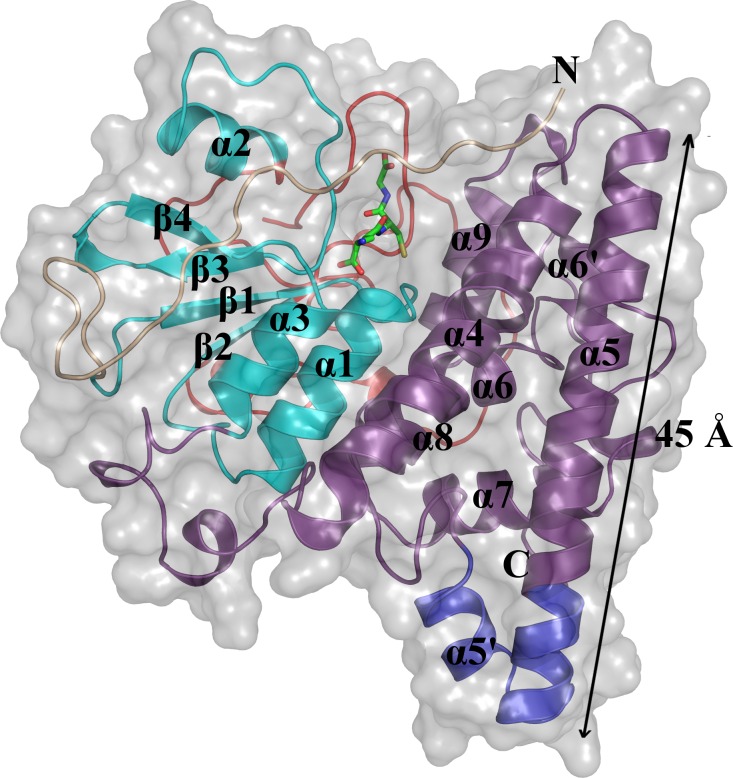
ScECM4 monomer with its specific features. The monomer of ScECM4 is represented as cartoon (N-terminal domain in cyan, C-terminal domain in violet). The N-terminal end is colored in wheat, the loop β2-α2 is colored in red and the extension of helix α5 along with helix α5’ are colored in blue.

**Fig 4:**
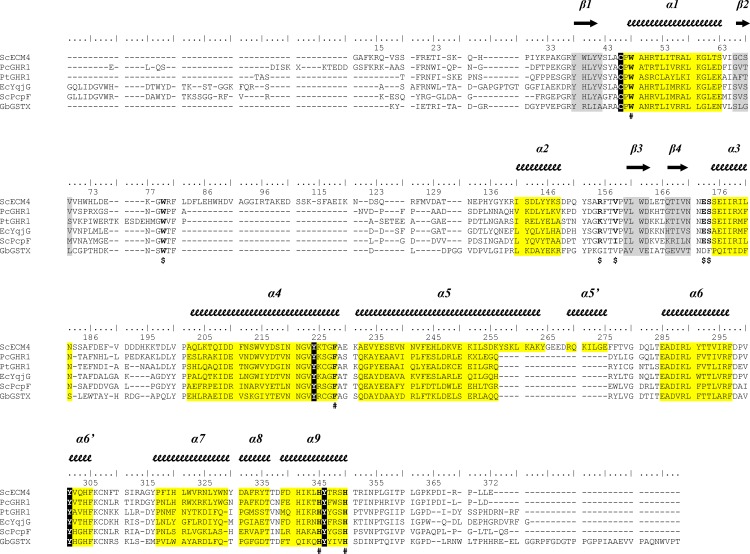
Structural alignment of the GSTXs available in the PDB and corresponding secondary structure elements. Structural alignment of 5 GSTXs available in the Protein Data Bank (PDB codes PcGHR1 3PPU, PtGHR1 4USS, EcYqjG 4G0K, ScPcpF 4FQU, GbGSTX 4PTS) with highlight of the secondary structure elements (helices in yellow, sheets in gray). Catalytic residues (the conserved catalytic cysteine and the triad of tyrosines) are highlighted in black. The symbols **$** indicate the positions of the residues that form interactions with the glutathione and the symbols **#** indicate the positions of the putative (hydro)quinone binding site residues. PtGHR1: GHR from *Populus trichocarpa*, PcGHR1: GHR1 from *Phanerochaete chrysosporium*, EcYqjG: YqjG from *Escherichia coli*, ScPcpF: PcpF from *Sphingobium chlorophenolicum*, GbGSTX: putative GHR from *Gordonia bronchialis*.

The structures of ScECM4 and ScECM4-SG were highly similar according to a RMSD of 0.120 Å between the two monomers. As expected, the monomer of ScECM4 exhibited the GST fold and dimerized with the pattern specific to GSTXs (Figs 1 and 3). The N-terminal domain (G11-K196) adopted the thioredoxin fold (β1α1β2α2β3β4α3) and the C-terminal domain (K197-E372) was found to be all helical (α4α5α5’α6α7α8α9). The typical features of GSTXs were also present: a long N-terminal end (37-residues), an extended loop between β2 and α2 (V70-R140) and a unique dimerization mode by the helical domain [2]. However, comparison of ScECM4 with other GSTX structures from the PDB highlighted some distinctive features detailed in the following lines (Table 2, Fig 4).

**Table 2:** Characterized GSTX structures in the Protein Data Bank (July 2016).

Available GSTX structures in the Protein Data Bank				
PDB code	Protein	Organism	Resolution (Å)	Ligands
5LKB, 5LKD	ScECM4	*Saccharomyces cerevisiae*	1.45	glutathione
3PPU	PcGHR1	*Phanerochaete chrysosporium*	2.3	glutathione
4USS	PtGHR1	*Populus trichocarpa*	2.5	glutathione
4G0I, 4G0L, 4G0K	EcYqjG	*Escherichia coli*	2.05	glutathione, GS-menadione
4FQU	ScPcpF	*Sphingobium chlorophenolicum*	3.0	none
4PTS	putative GHR	*Gordonia bronchialis*	2.83	none
3M1G	putative GHR	*Corynebacterium glutamicum*	2.1	none

The visible N-terminal end starting from G11 was found to be partially surrounding the active site in ScECM4 and in monomer A of ScECM4-SG. G11 was hydrogen-bonded with E230, at the C-terminal end of helix α4. The lack of electron density upstream G11 suggested a relative flexibility of the first residues. A similar position of the N-terminal end was described for monomer A of GHR1 from *P*. *chrysosporium* (PcGHR1) although in this case it was fully observed and covered the active site almost completely (S2A Fig). However, an N-terminal that surrounds the active site could be a particularity of fungal GHRs, since no similar fold was reported for the other GHRs. In the bacterial GHRs EcYqjG and ScPcpF, the N-terminal end adopted rather a characteristic β-hairpin conformation (S2B Fig) [14]. In poplar (PtGHR1), the N-terminal end adopted a more open conformation, although this end was not fully observed in the crystal structure [13].

The loop β2-α2 (residues V70-R140) was the second distinctiveness of ScECM4, and probably the most striking one. With 71 residues, it was far longer than the one of any other GSTXs (~30 residues; S2C Fig). As a comparison, it may be very shorter in non-Xi GSTs, such as in the human GST Omega 2 (6 residues). The usual conformation observed in the available models of GSTXs was a loop composed of three turns globally conserved. However variations were possible, such as a β-hairpin observed instead of the first turn in PtGHR1 (S2D Fig). In ScECM4, the first and the third turns were present. On the contrary, the second one was replaced by a long extension. It first deported the polypeptide chain from the core of the protein to the exterior side of the β-sheet opposite to the helix domain. Then, it returned towards the helix domain before it leaned on the partner monomer of the dimer. The side chain of S115 and the main chain of A117 were hydrogen-bonded to the side chain of E236 in the helix α5 of the other monomer (S2C Fig). To our knowledge, this was the first observation of a loop β2-α2 involved in the dimer interface in GSTs.

The third feature of ScECM4 was observed at helix α5 (A232-Y264). This helix was the largest secondary structure element (~45 Å), extending to the full height of the monomer (Fig 3). Indeed, its C-terminal end was elongated by two spiral turns with respect to the 6 ones usually observed in the other GSTX structures. Moreover, a little helix α5’ (R269-F275) absent in previous structures of GHRs was found after helix α5. α5’ redirected the polypeptide chain towards the protein core. Interestingly, such a helix was also found at the same place in EcYqjG and a putative GHR from *Gordonia bronchialis* but it was formed by residues situated at the end of the polypeptide sequence.

At last, we focused on D287 in helix α6 because it was shown to be crucial for the thiol-transferase activity, according to previous mutagenesis experiments [7]. This quasi-invariant aspartyl residue has a role in folding and stability [27] of human GST Pi and even in activity of human GST Alpha [28]. As observed in most known GST structures, D287 is a key residue in stabilizing helix α6 via an N-capping box [29]. In ScECM4, D287 was also hydrogen-bonded with 2 additional partners (T278 and W322), making it likely important for maintaining the tridimensional structures of the enzyme and thus its activity (S2E Fig).

*S*. *cerevisiae* harbors three homologous genes encoding for GSTs with a cysteine residue as the potential catalytic residue [7]. They were originally named Gto1, Gto2 (or ECM4) and Gto3 on the basis of their sequence homology with human GST Omega [7]. The present study demonstrated that ScECM4 rather was a GST Xi as most likely Gto1 and Gto3. These isoforms could reduce glutathionyl-(hydro)quinones as ScECM4 and/or could exhibit slightly different substrate spectrum since *S*. *cerevisiae* contained no GST Omega in its genome. A phylogenetic approach was used in order to evaluate whether the structural extension of ScECM4 (loop β2-α2 and helix α5) were found in other organisms (S3 Fig). Unambiguously, the analysis revealed that the elongations observed in ScECM4 were restricted to *Saccharomyces* and related genera (*Kazachstania*, *Zygosaccharomyces*, *Torulaspora*, and *Kluyveromyces)*. Interestingly a recent multigene phylogenetic analysis of the subphylum *Saccharomycotina* clustered these genera in the clade *Saccharomycetaceae* [30]. GSTX from yeast genera that did not belong to this clade did not show ScECM4 extensions such as *Candida albicans* (S3 Fig). The latter had only one gene encoding for a GSTX that displayed thioltransferase activity similar to ScECM4 [31]. Therefore, the ScECM4 structural extensions would have no influence on yeast GSTX activity.

### ScECM4 active site explained the ability of GHRs to reduce GS-(hydro)quinones

**Fig 5:**
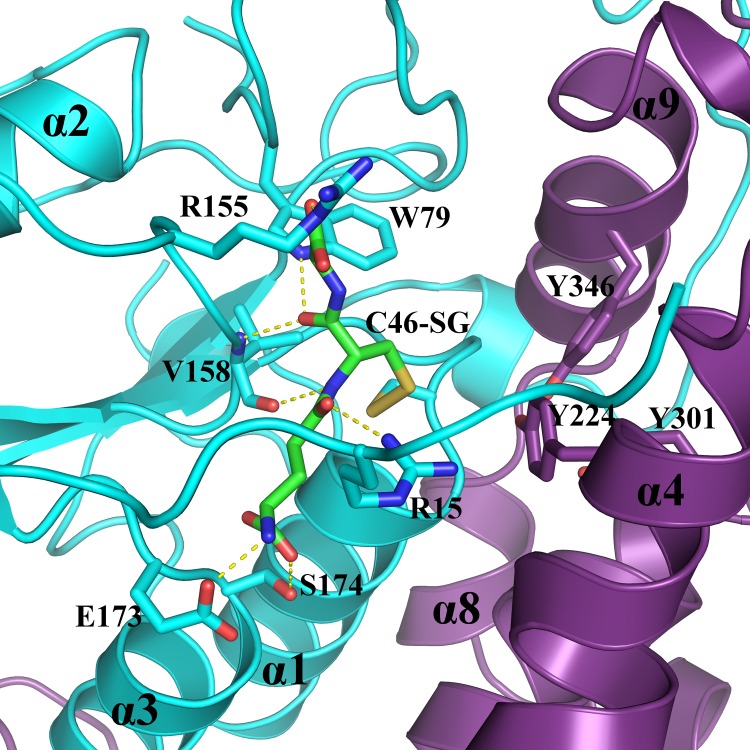
ScECM4 glutathione binding site. The catalytic cysteine C46, the residues involved in polar interactions with glutathione (GS) and the tyrosines Y224, Y301 and Y346 from the triad are represented as sticks. Polar interactions (yellow dashed lines) were observed between: E173 carboxylate group and GSH N-terminal amine group; NH of S174 and carboxylate group of GSH γ-glutamyl residue; S174 hydroxyl group and carboxylate group of GSH γ-glutamyl residue; R15 guanidinium group and carbonyl group of GSH γ-glutamyl residue; W79 indole group and carbonyl group of GSH cysteine; NHs and COs of V158 and of GSH cysteine; R155 guanidinium group and carboxylate group of GSH glycinyl residue.

GHRs are GSTs and as such classically exhibit a glutathionyl moiety binding site (G site) and a (hydro)quinone-moiety binding site (H site) [2]. Attempts to obtain crystals of complexes of ScECM4 with GS-menadione were not successful despite soaking or co-crystallization experiments. However, analysis at atomic resolution of Fo-Fc and 2Fo-Fc maps derived from these crystals allowed unambiguous identification of a GSH molecule in each monomer of ScECM4 (Fig 5). The sulfur atoms of GSH and C46 were found at a distance of 2.2 Å, indicating that the protein was covalently bound to GSH. C46 was previously identified as the catalytic cysteine of ScECM4 through monitoring the thiol-transferase activity [7]. These observations were consistent with activity observed with GS-menadione and described in the previous dedicated section. The backbones of V158, S174 and the side chains of R15, W79, R155, E173 and S174 were in direct interaction with GSH (see details in Fig 5 legend). Interestingly, the only conformational change between ScECM4 and its glutathionylated form was the side chain of R155 which stabilized the C-terminal moiety of GSH. This overall rigidity of the enzyme was already reported according to fluorescence emission experiments after mutation of the catalytic cysteine [7]. On the contrary, local conformational changes were described for the bacterial homolog EcYqjG upon binding with GSH or GS-menadione [14]. Our atomic resolution models of ScECM4 did not show such flexibility.

The position of GSH in the G-site of ScECM4 orientated the GSH cysteinyl side chain towards the hydroxyl groups of three tyrosines Y224 in α4, Y301 in α6’ and Y346 in α9 (Fig 5). These observations were consistent with the identification of a triad of tyrosine residues proposed to be involved in the catalytic mechanism of GHRs according to the bacterial model EcYqjG [14].

The structures of ScECM4 and ScECM4-SG raised questions about binding of GS-(hydro)quinones in the active site, and about the entailed catalytic mechanism. Unexpected absence of the menadione moiety in the active site did not allow precise description of the H site. Consequently, our analysis was based on the sole available structure of a complex between GHR and a glutathionylated compound. Overall superimposition of one monomer of EcYqjG in complex with GS-menadione (PDB code 4g0k, [14]) onto one monomer of ScECM4-SG resulted in satisfactory superposition of the residues that bordered the active site and of the glutathione (S4 Fig). Hence it provided a putative model of the way GS-menadione could bind in the active site of ScECM4.

**Fig 6:**
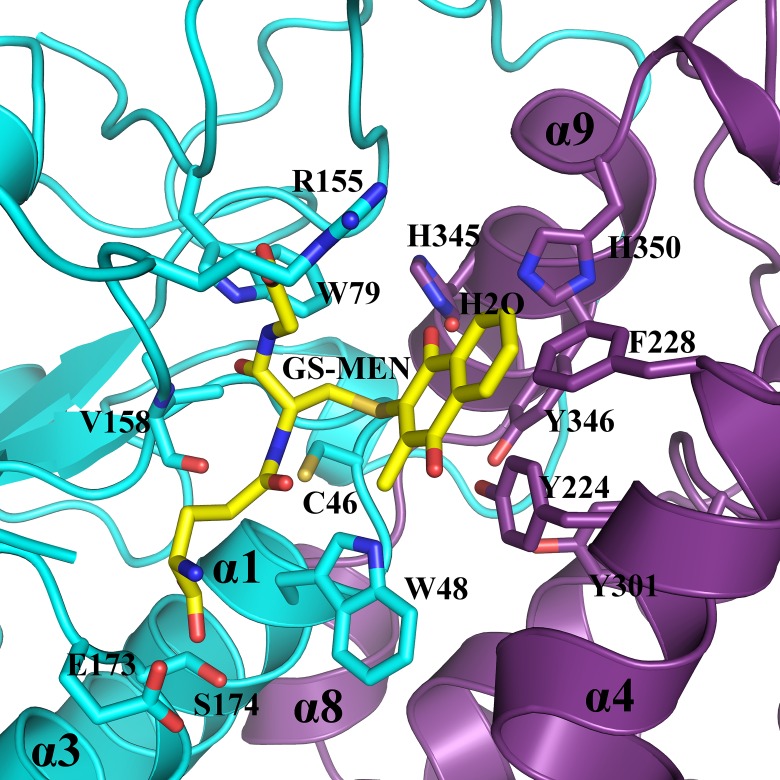
ScECM4 active site with superimposed GS-menadione. Residues of ScECM4 that form putative interactions with GS-menadione are labelled and represented as sticks. In addition to the G-site residues described in Fig 5, the drawing highlights the putative roles of W48, F228, H345, H350 and a water molecule (H_2_O) for the binding of the (hydro)quinone moiety, depending on its orientation.

Besides local rearrangements at the side chains of residues F14 and R15 to adapt to the presence of the menadione moiety of the substrate, most residues appeared in a suitable position to receive the substrate (Fig 6). The putative roles of five residues, F228, Y224, H345, H350 and W48 emerged from the active site analysis. As already proposed for EcYqjG, F228 could participate in substrate binding through π-stacking [14], however only in the case of voluminous polyaromatic substrates because of its remote position. The particular orientation of the side chain of Y195 almost parallel to the menadione could offer a similar interaction to smaller substrates. Furthermore, the dipole moment of its phenol group would be placed along the S-C bond of GS-menadione that was to be broken. It could induce an antiparallel dipole at this bond, enhancing reactivity of the GS-menadione sulfur towards the thiolate group of C46. Conserved in all known GHR structures, H345 and H350 from α9 formed hydrogen bonds with a water molecule. Although not modeled in EcYqjG, this water molecule was materialized at the same position by a 3.4σ peak in the corresponding Fo-Fc map. It interacted with the menadione carbonyl group that was positioned ortho to the glutathione. Such an interaction could be maintained with any (hydro)quinone that would orient this oxygen atom towards the glycyl end of GSH. Provided that some GS-(hydro)quinones could orient this oxygen atom towards the glutamyl end of GSH, an alternative interaction would be offered by the nitrogen of the indole group of W48, adequately positioned in all but EcYqjG GHR structures. In the latter, the high concentration of ammonium sulfate used to crystallize the enzyme probably explained the 180° rotation of the tryptophan side chain and therefore the lack of reactivity with GS-menadione. To summarize, H345 and H350, F228, Y224 from the conserved triad of tyrosines, and W48 that belonged to the sequence CPWA strictly conserved in GHRs could play crucial roles in binding of GS-(hydro)quinones, and in assisting C46 during catalysis.

## Conclusion

This study focused on the glutathionyl-(hydro)quinone reductase of *S*. *cerevisiae* named ScECM4. Two high resolution X-ray structures, with and without glutathione covalently bonded to the enzyme, were compared to previously known GHR structures. Besides the features that defined these GSTs as members of the class Xi, ScECM4 possessed a tremendously elongated loop between strand β2 and helix α2 involved in the dimer interface, two more spiral turns in helix α5 that make it 45 Å long and an additional helix α5’ with two helical turns. Phylogenetic analysis seemed to confine these specificities to GHRs of the genus saccharomyces and closely related genera. On the contrary, the enzyme possessed in its active site several residues that were conserved through all known GHR structures. They could provide GHRs with their reactivity towards GS-(hydro)quinones as observed both in solution and in the solid state. The present study supports the hypothesis deduced from previous biochemical and cytotoxicity studies that point to ScECM4 as a general protective factor involved in organism resistance to quinone toxicity.

## Supporting Information

S1 Fig Activity of ScECM4 with GS-menadione and various concentrations of GSH. UV/Vis spectrum of 100 μM GS-menadione with 1 μM ScECM4 was recorded after 10 min of reaction with various concentrations of GSH (0 to 1 mM).
(PDF)

S2 Fig Comparison of ScECM4 structure with known GHRs structures in the PDB. **A,** fungal GHRs structures with their N-terminal extensions bordering the active sites. N-terminal ends of ScECM4 (N-ter in violet, structure in light gray) and PcGHR1 (N-ter in cyan, structure in medium gray). The arrow indicates the position of the active site. **B,** bacterial GHR (EcYqjG) structure with its N-terminal extension colored in green. The arrow indicates the position of the active site. **C,** loop β2-α2 of ScECM4 (loop in blue, monomers in violet and cyan). Residues involved in polar contacts are labelled and represented as sticks. **D,** loop β2-α2 of PtGHR1 (loop in orange, monomer in salmon). **E,** N-capping residues of helix α6 of ScECM4. Residues involved in polar contacts are labelled and represented as sticks. In all panels, glutathione is represented as yellow sticks.
(PDF)

S3 Fig Multiple sequence alignment of ECM4-like proteins from yeasts. Sequence of *Candida albicans* CaGTO1 was retrieved from UniprotKB (UniprotKB ID: C4YL44). Sequences from yeasts except *Candida albicans* were retrieved from MycoCosm on the JGI website (JGI protein IDs are as follow: ScECM4, YKR076W; ScGTO1, YGR154C; ScGTO3, YMR251W; *Kazachstania africana* putative GST, KAFR_0H00400; *Zygosaccharomyces rouxii* putative GST ZYRO0B09922g; *Torulaspora delbrueckii* putative GST, TDEL_0B02560; *Kluyveromyces lactis* putative GST, KLLA0F12056g; *Pichia pastoris* putative GST, PAS_chr2-1_03). Sequences were aligned using Promals3D with the structure of ScECM4 as input and were manually annotated. Catalytic residues (the conserved catalytic cysteine and the tyrosines of the triad) are colored in white and highlighted in black. The symbols **$** indicate the positions of the residues that form interactions with the glutathione and the symbols **#** indicate the positions of the putative (hydro)quinone binding site residues. ScECM4 specific extensions are highlighted in yellow.
(PDF)

S4 Fig Superimposed active sites of EcYqjG and ScECM4. EcYqjG structure in complex with GS-menadione was superimposed onto ScECM4-SG structure in order to identify residues that form putative interactions with the (hydro)quinone moiety. Residues of both active sites, along with the glutathionyl moieties showed good superimposition, thus allowing the identification of ScECM4 putative H site residues. ScECM4-SG is colored in cyan (N-terminal domain) and violet (C-terminal domain) with GSH as green sticks, EcYqjG is colored in tints of gray with GS-menadione as yellow sticks.
(PDF)

S1 File PDB Validation report for ScECM4 (PDF)

S2 File PDB Validation report for ScECM4-SG (PDF)
