# Supplementary material for: Crystal Structure of Saccharomyces cerevisiae ECM4, a Xi-Class Glutathione Transferase that Reacts with Glutathionyl-(hydro)quinones
Source: PLoS One. 2016 Oct 13;11(10):e0164678. doi: 10.1371/journal.pone.0164678 (PMC5063366; doi:10.1371/journal.pone.0164678)
Supplement: S1 Fig — UV/Vis spectrum of 100 μM GS-menadione with 1 μM ScECM4 was recorded after 10 min of reaction with various concentrations of GSH (0 to 1 mM). (PDF) [file pone.0164678.s001.pdf]

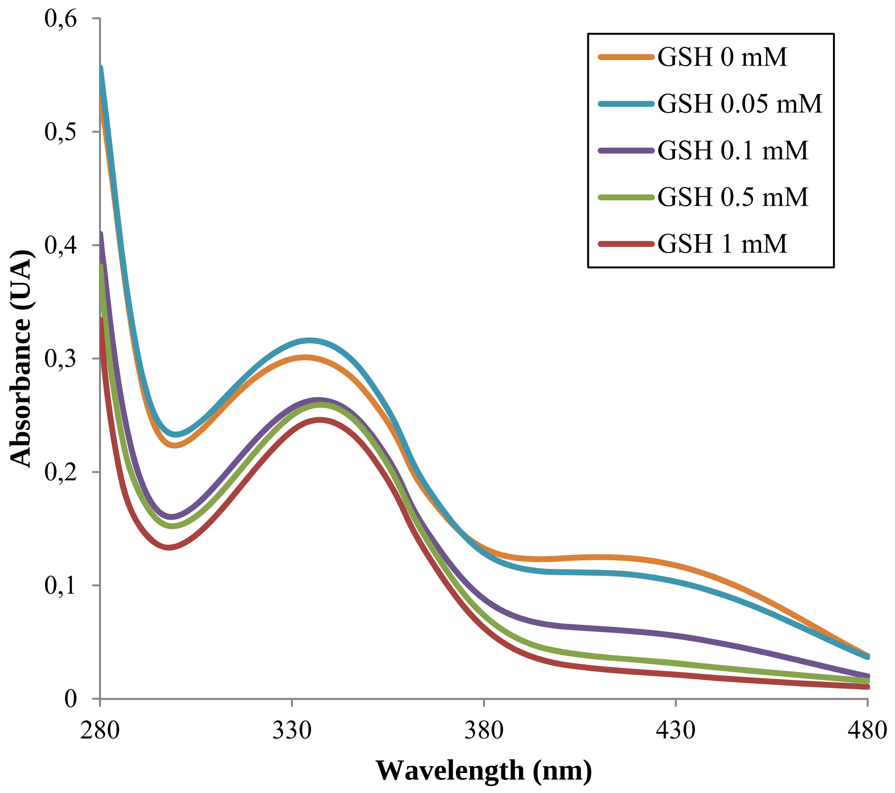

Figure S1. Activity of ScECM4 with GS-menadione and various concentrations of GSH

UV/Vis spectrum of 100 µM GS-menadione with 1 µM ScECM4 was recorded after 10 minutes reaction with various concentrations of GSH (0 to 1 mM).
