# Supplementary material for: Crystal Structure of Saccharomyces cerevisiae ECM4, a Xi-Class Glutathione Transferase that Reacts with Glutathionyl-(hydro)quinones
Source: PLoS One. 2016 Oct 13;11(10):e0164678. doi: 10.1371/journal.pone.0164678 (PMC5063366; doi:10.1371/journal.pone.0164678)
Supplement: S2 Fig — A, fungal GHRs structures with their N-terminal extensions bordering the active sites. N-terminal ends of ScECM4 (N-ter in violet, structure in light gray) and PcGHR1 (N-ter in cyan, structure in medium gray). The arrow indicates the position of the active site. B, bacterial GHR (EcYqjG) structure with its N-terminal extension colored in green. The arrow indicates the position of the active site. C, loop β2-α2 of ScECM4 (loop in blue, monomers in violet and cyan). Residues involved in polar contacts are labelled and represented as sticks. D, loop β2-α2 of PtGHR1 (loop in orange, monomer in salmon). E, N-capping residues of helix α6 of ScECM4. Residues involved in polar contacts are labelled and represented as sticks. In all panels, glutathione is represented as yellow sticks. (PDF) [file pone.0164678.s002.pdf]

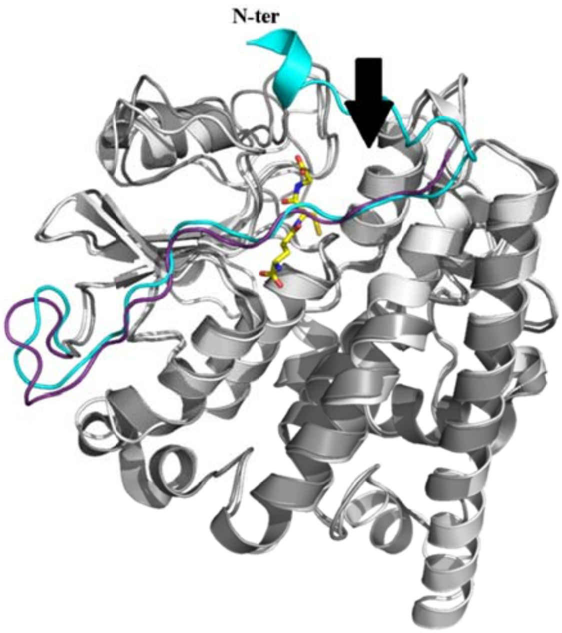

A.

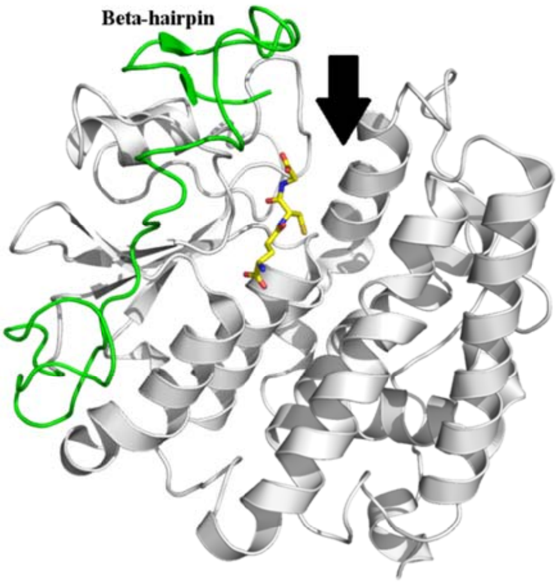

B.

D.

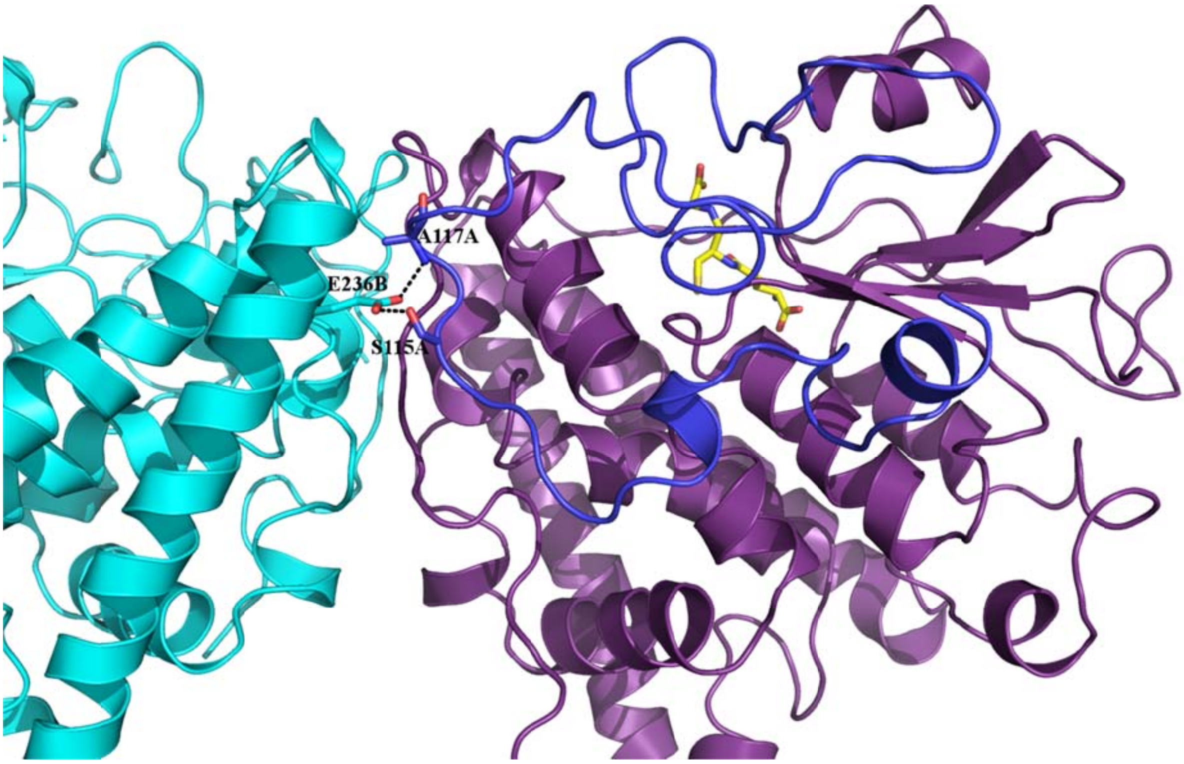

C.

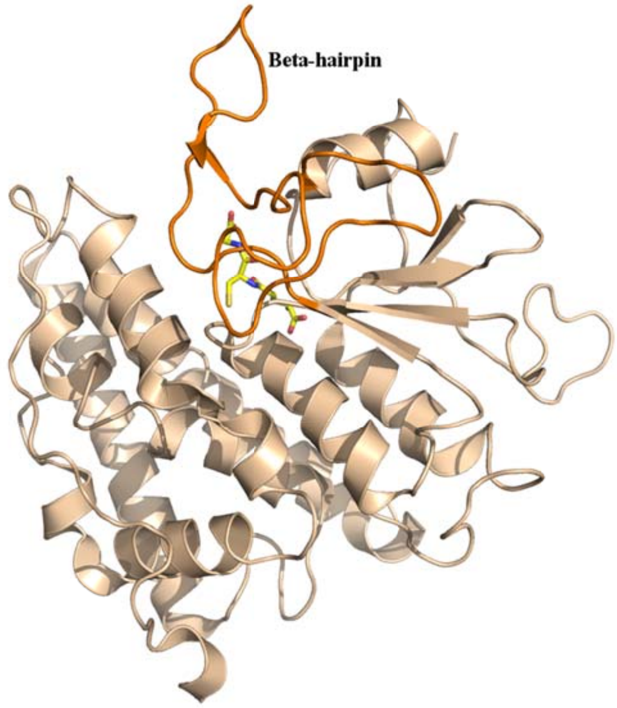

D.

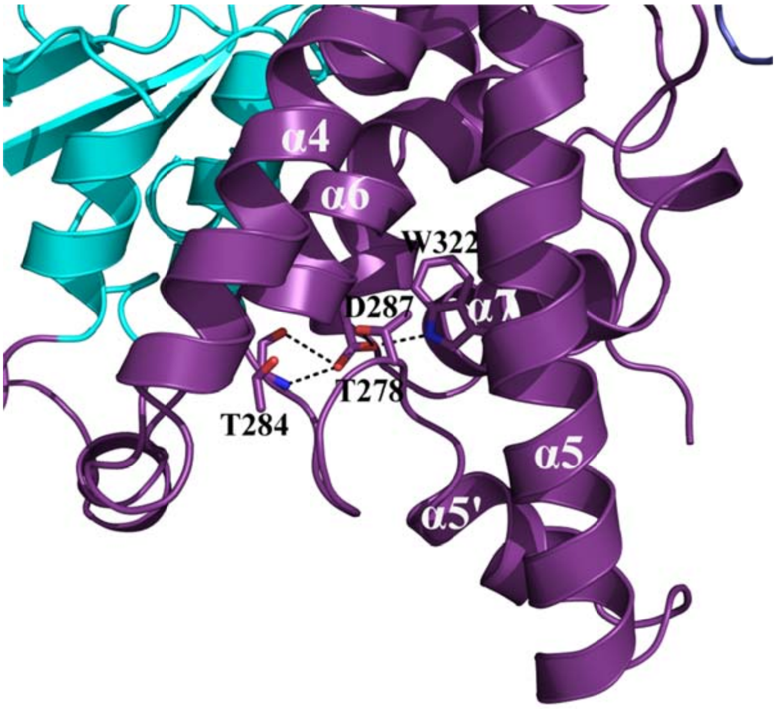

E.

Fig S2. Comparison of ScECM4 structure with known GHRs structures in the PDB

A, fungal GHRs structures with their N-terminal extensions bordering the active sites. N-terminal ends of ScECM4 (N-ter in violet, structure in light gray) and PcGHR1 (N-ter in cyan, structure in medium gray). The arrow indicates the position of the active site. **B,** bacterial GHR (EcYqjG) structure with its N-terminal extension colored in green. The arrow indicates the position of the active site. **C,** loop β2-α2 of ScECM4 (loop in blue, monomers in violet and cyan). Residues involved in polar contacts are labelled and represented as sticks. **D,** loop β2-α2 of PtGHR1 (loop in orange, monomer in salmon). **E,** N-capping residues of helix α6 of ScECM4. Residues involved in polar contacts are labelled and represented as sticks. In all panels, glutathione is represented as yellow sticks.
