# Supplementary material for: Crystal Structure of Saccharomyces cerevisiae ECM4, a Xi-Class Glutathione Transferase that Reacts with Glutathionyl-(hydro)quinones
Source: PLoS One. 2016 Oct 13;11(10):e0164678. doi: 10.1371/journal.pone.0164678 (PMC5063366; doi:10.1371/journal.pone.0164678)
Supplement: S3 Fig — Sequence of Candida albicans CaGTO1 was retrieved from UniprotKB (UniprotKB ID: C4YL44). Sequences from yeasts except Candida albicans were retrieved from MycoCosm on the JGI website (JGI protein IDs are as follow: ScECM4, YKR076W; ScGTO1, YGR154C; ScGTO3, YMR251W; Kazachstania africana putative GST, KAFR_0H00400; Zygosaccharomyces rouxii putative GST ZYRO0B09922g; Torulaspora delbrueckii putative GST, TDEL_0B02560; Kluyveromyces lactis putative GST, KLLA0F12056g; Pichia pastoris putative GST, PAS_chr2-1_03). Sequences were aligned using Promals3D with the structure of ScECM4 as input and were manually annotated. Catalytic residues (the conserved catalytic cysteine and the tyrosines of the triad) are colored in white and highlighted in black. The symbols $ indicate the positions of the residues that form interactions with the glutathione and the symbols # indicate the positions of the putative (hydro)quinone binding site residues. ScECM4 specific extensions are highlighted in yellow. (PDF) [file pone.0164678.s003.pdf]

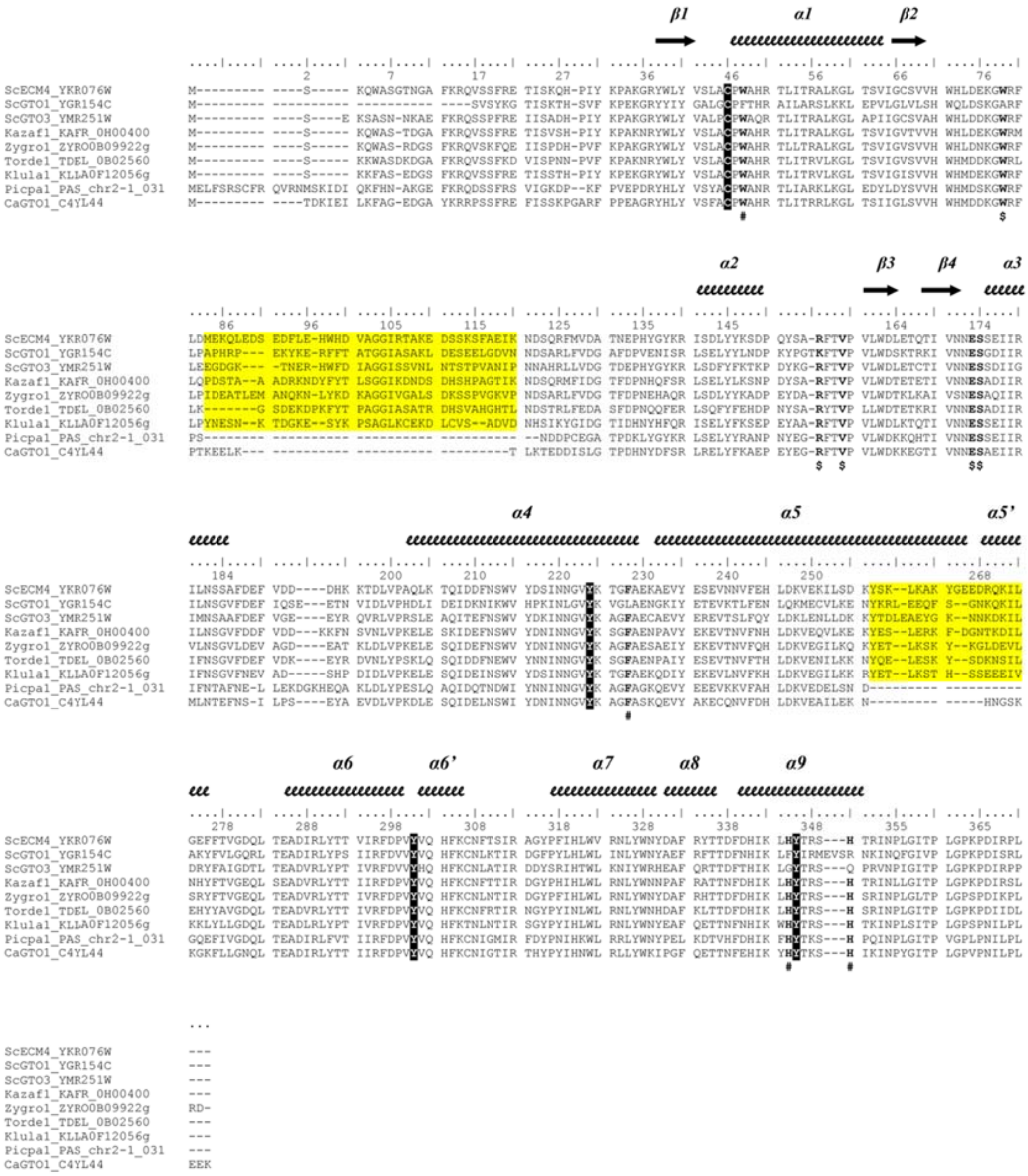

Fig S3. Multiple sequence alignment of ECM4-like proteins from yeasts.

Sequence of *Candida albicans* CaGTO1 was retrieved from UniprotKB (UniprotKB ID: C4YL44). Sequences from yeasts except *Candida albicans* were retrieved from MycoCosm on the JGI website (JGI protein IDs are as follow: ScECM4, YKR076W; ScGTO1, YGR154C; ScGTO3, YMR251W; *Kazachstania africana* putative GST, KAFR_0H00400; *Zygosaccharomyces rouxii* putative GST ZYRO0B09922g; *Torulaspora delbrueckii* putative GST, TDEL_0B02560; *Kluyveromyces lactis* putative GST, KLLA0F12056g; *Pichia pastoris* putative GST, PAS_chr2-1_03). Sequences were aligned using Promals3D with the structure of ScECM4 as input and were manually annotated. Catalytic residues (the conserved catalytic cysteine and the tyrosines of the triad) are colored in white and highlighted in black. The symbols **$** indicate the positions of the residues that form interactions with the glutathione and the symbols **#** indicate the positions of the putative (hydro)quinone binding site residues. ScECM4 specific extensions are highlighted in yellow.
