# Supplementary material for: Crystal Structure of Saccharomyces cerevisiae ECM4, a Xi-Class Glutathione Transferase that Reacts with Glutathionyl-(hydro)quinones
Source: PLoS One. 2016 Oct 13;11(10):e0164678. doi: 10.1371/journal.pone.0164678 (PMC5063366; doi:10.1371/journal.pone.0164678)
Supplement: S4 Fig — EcYqjG structure in complex with GS-menadione was superimposed onto ScECM4-SG structure in order to identify residues that form putative interactions with the (hydro)quinone moiety. Residues of both active sites, along with the glutathionyl moieties showed good superimposition, thus allowing the identification of ScECM4 putative H site residues. ScECM4-SG is colored in cyan (N-terminal domain) and violet (C-terminal domain) with GSH as green sticks, EcYqjG is colored in tints of gray with GS-menadione as yellow sticks. (PDF) [file pone.0164678.s004.pdf]

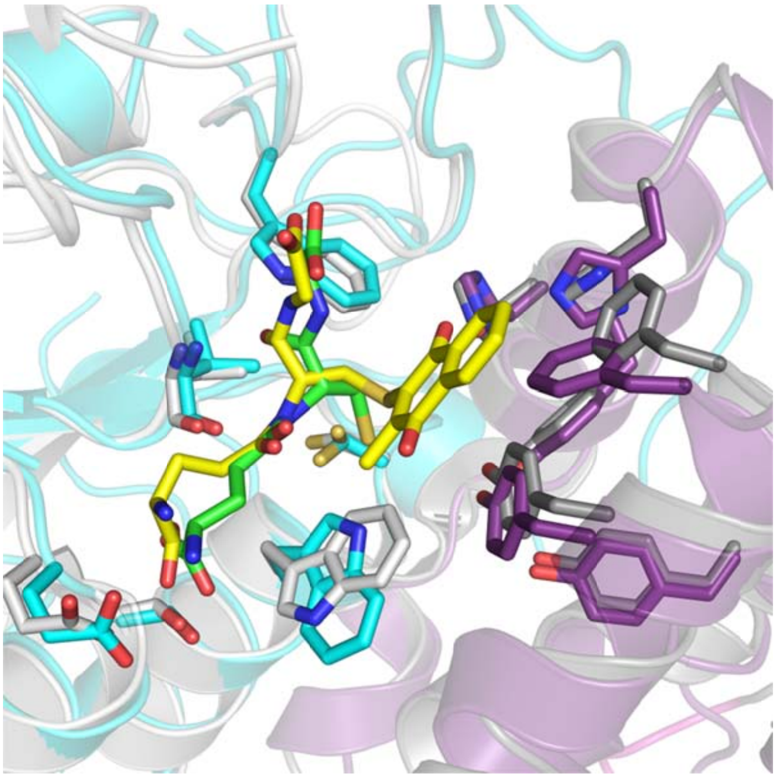

Fig S4. Superimposed active sites of EcYqjG and ScECM4.

EcYqjG structure in complex with GS-menadione was superimposed onto ScECM4-SG structure in order to identify residues that form putative interactions with the (hydro)quinone moiety. Residues of both active sites, along with the glutathionyl moieties showed good superimposition, thus allowing the identification of ScECM4 putative H site residues. ScECM4-SG is colored in cyan (N-terminal domain) and violet (C-terminal domain) with GSH as green sticks, EcYqjG is colored in tints of gray with GS-menadione as yellow sticks.
