# Supplementary material for: Crystal Structure of Saccharomyces cerevisiae ECM4, a Xi-Class Glutathione Transferase that Reacts with Glutathionyl-(hydro)quinones
Source: PLoS One. 2016 Oct 13;11(10):e0164678. doi: 10.1371/journal.pone.0164678 (PMC5063366; doi:10.1371/journal.pone.0164678)
Supplement: S1 File — (PDF) [file pone.0164678.s005.pdf]

wwPDB X-ray Structure Validation Summary Report (i)

Jul 22, 2016 – 03:34 PM BST

PDB ID	:	5LKB
Title	:	Crystal structure of the Xi glutathione transferase ECM4 from Saccharomyces cerevisiae
Authors	:	Schwartz, M.; Didierjean, C.; Hecker, A.; Girardet, J.M.; Morel-Rouhier, M.; Gelhaye, E.; Favier, F.
Deposited on	:	2016-07-22
Resolution	:	1.45 Å(reported)

This is a wwPDB X-ray Structure Validation Summary Report.
We welcome your comments at validation@mail.wwpdb.org
A user guide is available at
http://wwpdb.org/validation/2016/XrayValidationReportHelp
with specific help available everywhere you see the (i) symbol.

CONFIDENTIAL VALIDATION REPORT

The following versions of software and data (see references (i)) were used in the production of this report:

MolProbity	:	4.02b-467
Mogul	:	1.7.1 (RC1), CSD as537be (2016)
Xtriage (Phenix)	:	1.9-1692
EDS	:	rb-20027790
Percentile statistics	:	20151230.v01 (using entries in the PDB archive December 30th 2015)
Refmac	:	5.8.0135
CCP4	:	6.5.0
Ideal geometry (proteins)	:	Engh & Huber (2001)
Ideal geometry (DNA, RNA)	:	Parkinson et al. (1996)
Validation Pipeline (wwPDB-VP)	:	rb-20027790

1 Overall quality at a glance (i)

The following experimental techniques were used to determine the structure:
X-RAY DIFFRACTION

The reported resolution of this entry is 1.45 Å.

Percentile scores (ranging between 0-100) for global validation metrics of the entry are shown in the following graphic. The table shows the number of entries on which the scores are based.

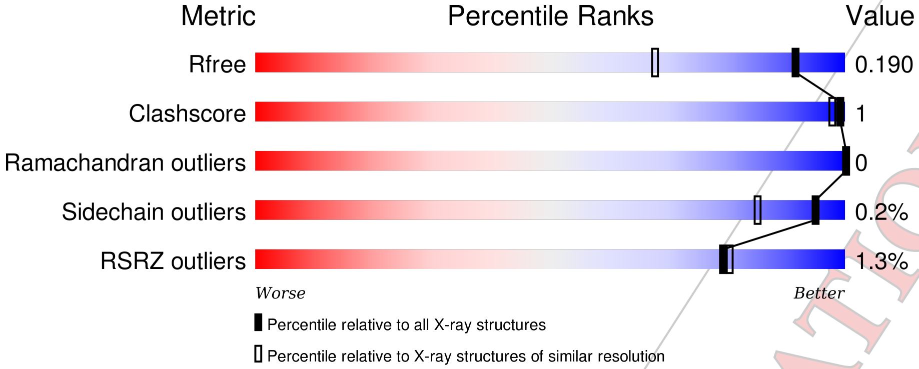

Metric	Whole archive (#Entries)	Similar resolution (#Entries, resolution range(Å))
R_{free}	91344	1278 (1.48-1.44)
Clashscore	102246	1336 (1.48-1.44)
Ramachandran outliers	100387	1320 (1.48-1.44)
Sidechain outliers	100360	1320 (1.48-1.44)
RSRZ outliers	91569	1279 (1.48-1.44)

The table below summarises the geometric issues observed across the polymeric chains and their fit to the electron density. The red, orange, yellow and green segments on the lower bar indicate the fraction of residues that contain outliers for >=3, 2, 1 and 0 types of geometric quality criteria. A grey segment represents the fraction of residues that are not modelled. The numeric value for each fraction is indicated below the corresponding segment, with a dot representing fractions <=5% The upper red bar (where present) indicates the fraction of residues that have poor fit to the electron density. The numeric value is given above the bar.

Mol	Chain	Length	Quality of chain
1	A	378	2% / 88% • 9%
1	B	378	88% • 9%

The following table lists non-polymeric compounds, carbohydrate monomers and non-standard residues in protein, DNA, RNA chains that are outliers for geometric or electron-density-fit criteria:

CONFIDENTIAL VALIDATION REPORT

Mol	Type	Chain	Res	Chirality	Geometry	Clashes	Electron density
2	GOL	B	401	-	-	-	X

CONFIDENTIAL VALIDATION REPORT

2 Entry composition (i)

There are 3 unique types of molecules in this entry. The entry contains 12108 atoms, of which 5571 are hydrogens and 0 are deuteriums.

In the tables below, the ZeroOcc column contains the number of atoms modelled with zero occupancy, the AltConf column contains the number of residues with at least one atom in alternate conformation and the Trace column contains the number of residues modelled with at most 2 atoms.

- Molecule 1 is a protein called Glutathione S-transferase omega-like 2.

Mol	Chain	Residues	Atoms						ZeroOcc	AltConf	Trace
			Total	C	H	N	O	S			
1	A	344	5660	1854	2788	489	525	4	126	2	0
1	B	344	5645	1846	2783	490	522	4	181	1	0

There are 16 discrepancies between the modelled and reference sequences:

Chain	Residue	Modelled	Actual	Comment	Reference
A	371	LEU	-	expression tag	UNP P36156
A	372	GLU	-	expression tag	UNP P36156
A	373	HIS	-	expression tag	UNP P36156
A	374	HIS	-	expression tag	UNP P36156
A	375	HIS	-	expression tag	UNP P36156
A	376	HIS	-	expression tag	UNP P36156
A	377	HIS	-	expression tag	UNP P36156
A	378	HIS	-	expression tag	UNP P36156
B	371	LEU	-	expression tag	UNP P36156
B	372	GLU	-	expression tag	UNP P36156
B	373	HIS	-	expression tag	UNP P36156
B	374	HIS	-	expression tag	UNP P36156
B	375	HIS	-	expression tag	UNP P36156
B	376	HIS	-	expression tag	UNP P36156
B	377	HIS	-	expression tag	UNP P36156
B	378	HIS	-	expression tag	UNP P36156

- Molecule 2 is GLYCEROL (three-letter code: GOL) (formula: $C_3H_8O_3$).

CONFIDENTIAL VALIDATION REPORT

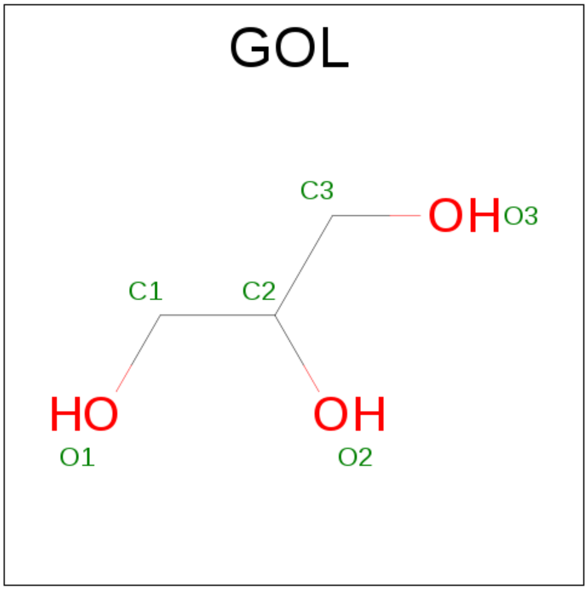

Mol	Chain	Residues	Atoms	ZeroOcc	AltConf
2	A	1	Total C O<br>6 3 3	0	0
2	A	1	Total C O<br>6 3 3	0	0
2	B	1	Total C O<br>6 3 3	0	0

- Molecule 3 is water.

Mol	Chain	Residues	Atoms	ZeroOcc	AltConf
3	A	403	Total O<br>403 403	0	0
3	B	382	Total O<br>382 382	0	0

CONFIDENTIAL VALIDATION REPORT

3 Residue-property plots (i)

These plots are drawn for all protein, RNA and DNA chains in the entry. The first graphic for a chain summarises the proportions of errors displayed in the second graphic. The second graphic shows the sequence view annotated by issues in geometry and electron density. Residues are color-coded according to the number of geometric quality criteria for which they contain at least one outlier: green = 0, yellow = 1, orange = 2 and red = 3 or more. A red dot above a residue indicates a poor fit to the electron density (RSRZ > 2). Stretches of 2 or more consecutive residues without any outlier are shown as a green connector. Residues present in the sample, but not in the model, are shown in grey.

- Molecule 1: Glutathione S-transferase omega-like 2

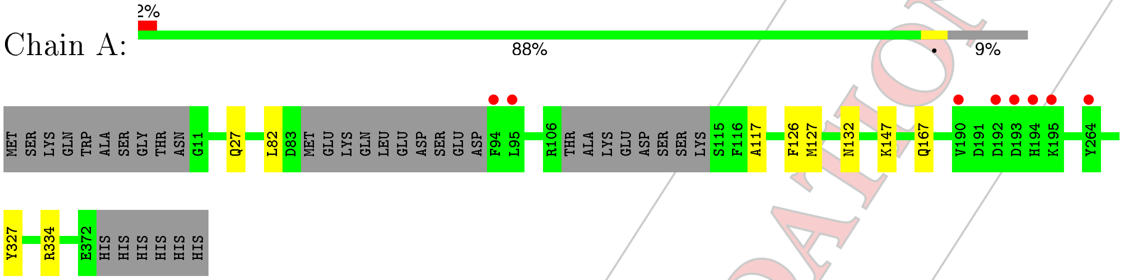

- Molecule 1: Glutathione S-transferase omega-like 2

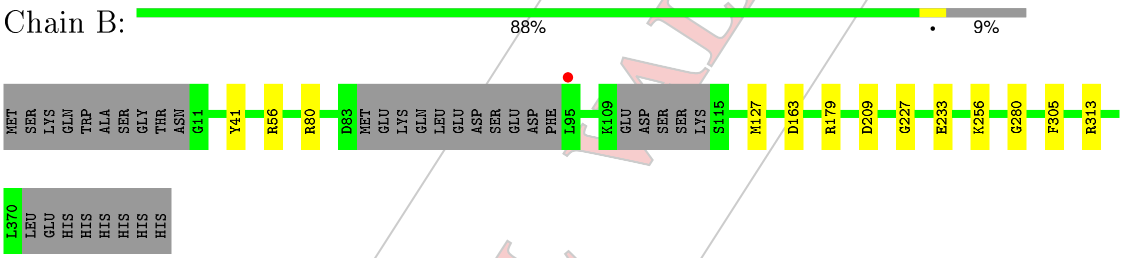

CONFIDENTIAL VALIDATION REPORT

4 Data and refinement statistics (i)

Property	Value	Source
Space group	P 1 21 1	Depositor
Cell constants<br>a, b, c, α, β, γ	55.51Å 82.96Å 80.65Å<br>90.00° 95.13° 90.00°	Depositor
Resolution (Å)	33.97 – 1.45<br>46.01 – 1.45	Depositor<br>EDS
% Data completeness<br>(in resolution range)	96.4 (33.97-1.45)<br>88.6 (46.01-1.45)	Depositor<br>EDS
R_{merge}	(Not available)	Depositor
R_{sym}	(Not available)	Depositor
$< I/\sigma(I) >$ [1]	1.93 (at 1.45Å)	Xtriage
Refinement program	PHENIX (1.10.1_2155: ???)	Depositor
R, R_{free}	0.167 , 0.194<br>0.164 , 0.190	Depositor<br>DCC
R_{free} test set	5722 reflections (5.02%)	DCC
Wilson B-factor (Å^2)	15.1	Xtriage
Anisotropy	0.706	Xtriage
Bulk solvent k_{sol}(e/Å^3), B_{sol}(Å^2)	0.44 , 53.6	EDS
L-test for twinning[2]	$< \|L\| >$ = 0.50, $< L^2 >$ = 0.33	Xtriage
Estimated twinning fraction	No twinning to report.	Xtriage
F_o,F_c correlation	0.97	EDS
Total number of atoms	12108	wwPDB-VP
Average B, all atoms (Å^2)	24.0	wwPDB-VP

Xtriage's analysis on translational NCS is as follows: *The largest off-origin peak in the Patterson function is 5.45% of the height of the origin peak. No significant pseudotranslation is detected.*

[1] Intensities estimated from amplitudes.

[2] Theoretical values of $< |L| >$, $< L^2 >$ for acentric reflections are 0.5, 0.333 respectively for untwinned datasets, and 0.375, 0.2 for perfectly twinned datasets.

CONFIDENTIAL VALIDATION REPORT

5 Model quality (i)

5.1 Standard geometry (i)

Bond lengths and bond angles in the following residue types are not validated in this section: GOL

The Z score for a bond length (or angle) is the number of standard deviations the observed value is removed from the expected value. A bond length (or angle) with $|Z| > 5$ is considered an outlier worth inspection. RMSZ is the root-mean-square of all Z scores of the bond lengths (or angles).

Mol	Chain	Bond lengths		Bond angles	
		RMSZ	#$|Z|$ >5	RMSZ	#$|Z|$ >5
1	A	0.73	0/2961	0.81	0/4017
1	B	0.78	1/2947 (0.0%)	0.87	8/3998 (0.2%)
All	All	0.76	1/5908 (0.0%)	0.84	8/8015 (0.1%)

All (1) bond length outliers are listed below:

Mol	Chain	Res	Type	Atoms	Z	Observed(Å)	Ideal(Å)
1	B	41	TYR	CE1-CZ	-5.92	1.30	1.38

The worst 5 of 8 bond angle outliers are listed below:

Mol	Chain	Res	Type	Atoms	Z	Observed(^{o})	Ideal(^{o})
1	B	179	ARG	NE-CZ-NH1	7.07	123.83	120.30
1	B	313	ARG	NE-CZ-NH1	6.48	123.54	120.30
1	B	80	ARG	NE-CZ-NH1	-6.23	117.19	120.30
1	B	313	ARG	NE-CZ-NH2	-5.70	117.45	120.30
1	B	127	MET	CG-SD-CE	-5.58	91.28	100.20

There are no chirality outliers.

There are no planarity outliers.

5.2 Too-close contacts (i)

In the following table, the Non-H and H(model) columns list the number of non-hydrogen atoms and hydrogen atoms in the chain respectively. The H(added) column lists the number of hydrogen atoms added and optimized by MolProbity. The Clashes column lists the number of clashes within the asymmetric unit, whereas Symm-Clashes lists symmetry related clashes.

CONFIDENTIAL VALIDATION REPORT

Mol	Chain	Non-H	H(model)	H(added)	Clashes	Symm-Clashes
1	A	2872	2788	2786	5	0
1	B	2862	2783	2782	3	0
2	A	12	0	16	0	0
2	B	6	0	8	0	0
3	A	403	0	0	0	0
3	B	382	0	0	0	0
All	All	6537	5571	5592	7	0

The all-atom clashscore is defined as the number of clashes found per 1000 atoms (including hydrogen atoms). The all-atom clashscore for this structure is 1.

The worst 5 of 7 close contacts within the same asymmetric unit are listed below, sorted by their clash magnitude.

Atom-1	Atom-2	Interatomic distance (Å)	Clash overlap (Å)
1:A:147:LYS:HE2	1:A:167:GLN:OE1	2.08	0.53
1:A:327:TYR:O	1:A:334:ARG:HB2	2.16	0.46
1:B:256:LYS:NZ	1:B:280:GLY:O	2.50	0.44
1:A:117:ALA:HB1	1:B:233:GLU:HG3	1.99	0.43
1:A:126:PHE:CD2	1:A:127:MET:HG2	2.54	0.43

There are no symmetry-related clashes.

5.3 Torsion angles (i)

5.3.1 Protein backbone (i)

In the following table, the Percentiles column shows the percent Ramachandran outliers of the chain as a percentile score with respect to all X-ray entries followed by that with respect to entries of similar resolution.

The Analysed column shows the number of residues for which the backbone conformation was analysed, and the total number of residues.

Mol	Chain	Analysed	Favoured	Allowed	Outliers	Percentiles	
1	A	340/378 (90%)	334 (98%)	6 (2%)	0	100	100
1	B	339/378 (90%)	334 (98%)	5 (2%)	0	100	100
All	All	679/756 (90%)	668 (98%)	11 (2%)	0	100	100

There are no Ramachandran outliers to report.

CONFIDENTIAL VALIDATION REPORT

5.3.2 Protein sidechains (i)

In the following table, the Percentiles column shows the percent sidechain outliers of the chain as a percentile score with respect to all X-ray entries followed by that with respect to entries of similar resolution.

The Analysed column shows the number of residues for which the sidechain conformation was analysed, and the total number of residues.

Mol	Chain	Analysed	Rotameric	Outliers	Percentiles	
1	A	313/342 (92%)	312 (100%)	1 (0%)	94	84
1	B	311/342 (91%)	311 (100%)	0	100	100
All	All	624/684 (91%)	623 (100%)	1 (0%)	95	85

All (1) residues with a non-rotameric sidechain are listed below:

Mol	Chain	Res	Type
1	A	27	GLN

Some sidechains can be flipped to improve hydrogen bonding and reduce clashes. There are no such sidechains identified.

5.3.3 RNA (i)

There are no RNA molecules in this entry.

5.4 Non-standard residues in protein, DNA, RNA chains (i)

There are no non-standard protein/DNA/RNA residues in this entry.

5.5 Carbohydrates (i)

There are no carbohydrates in this entry.

5.6 Ligand geometry (i)

3 ligands are modelled in this entry.

In the following table, the Counts columns list the number of bonds (or angles) for which Mogul statistics could be retrieved, the number of bonds (or angles) that are observed in the model and the number of bonds (or angles) that are defined in the chemical component dictionary. The Link column lists molecule types, if any, to which the group is linked. The Z score for a bond length

CONFIDENTIAL VALIDATION REPORT

(or angle) is the number of standard deviations the observed value is removed from the expected value. A bond length (or angle) with $|Z| > 2$ is considered an outlier worth inspection. RMSZ is the root-mean-square of all Z scores of the bond lengths (or angles).

Mol	Type	Chain	Res	Link	Bond lengths			Bond angles		
					Counts	RMSZ	$\#\|Z\| > 2$	Counts	RMSZ	$\#\|Z\| > 2$
2	GOL	A	401	-	5,5,5	0.48	0	5,5,5	0.45	0
2	GOL	A	402	-	5,5,5	0.27	0	5,5,5	0.35	0
2	GOL	B	401	-	5,5,5	0.37	0	5,5,5	1.03	1 (20%)

In the following table, the Chirals column lists the number of chiral outliers, the number of chiral centers analysed, the number of these observed in the model and the number defined in the chemical component dictionary. Similar counts are reported in the Torsion and Rings columns. '-' means no outliers of that kind were identified.

Mol	Type	Chain	Res	Link	Chirals	Torsions	Rings
2	GOL	A	401	-	-	0/4/4/4	0/0/0/0
2	GOL	A	402	-	-	0/4/4/4	0/0/0/0
2	GOL	B	401	-	-	0/4/4/4	0/0/0/0

There are no bond length outliers.

All (1) bond angle outliers are listed below:

Mol	Chain	Res	Type	Atoms	Z	Observed(^{o})	Ideal(^{o})
2	B	401	GOL	C3-C2-C1	-2.08	102.31	111.06

There are no chirality outliers.

There are no torsion outliers.

There are no ring outliers.

No monomer is involved in short contacts.

5.7 Other polymers (i)

There are no such residues in this entry.

5.8 Polymer linkage issues (i)

There are no chain breaks in this entry.

CONFIDENTIAL VALIDATION REPORT

6 Fit of model and data (i)

6.1 Protein, DNA and RNA chains (i)

In the following table, the column labelled '#RSRZ> 2' contains the number (and percentage) of RSRZ outliers, followed by percent RSRZ outliers for the chain as percentile scores relative to all X-ray entries and entries of similar resolution. The OWAB column contains the minimum, median, 95th percentile and maximum values of the occupancy-weighted average B-factor per residue. The column labelled 'Q< 0.9' lists the number of (and percentage) of residues with an average occupancy less than 0.9.

Mol	Chain	Analysed	<RSRZ>	#RSRZ>2			OWAB(Å^2)	Q<0.9
1	A	344/378 (91%)	-0.34	8 (2%)	64	65	13, 21, 39, 56	15 (4%)
1	B	344/378 (91%)	-0.38	1 (0%)	94	96	12, 18, 36, 55	18 (5%)
All	All	688/756 (91%)	-0.36	9 (1%)	79	80	12, 20, 38, 56	33 (4%)

The worst 5 of 9 RSRZ outliers are listed below:

Mol	Chain	Res	Type	RSRZ
1	A	94	PHE	4.0
1	A	195	LYS	2.8
1	A	190	VAL	2.7
1	A	193	ASP	2.6
1	A	95	LEU	2.4

6.2 Non-standard residues in protein, DNA, RNA chains (i)

There are no non-standard protein/DNA/RNA residues in this entry.

6.3 Carbohydrates (i)

There are no carbohydrates in this entry.

6.4 Ligands (i)

In the following table, the Atoms column lists the number of modelled atoms in the group and the number defined in the chemical component dictionary. LLDF column lists the quality of electron density of the group with respect to its neighbouring residues in protein, DNA or RNA chains. The B-factors column lists the minimum, median, 95th percentile and maximum values of B factors of atoms in the group. The column labelled 'Q< 0.9' lists the number of atoms with occupancy less than 0.9.

CONFIDENTIAL VALIDATION REPORT

Mol	Type	Chain	Res	Atoms	RSCC	RSR	LLDF	B-factors($Å^2$)	Q<0.9
2	GOL	B	401	6/6	0.93	0.13	5.16	20,25,28,34	0
2	GOL	A	402	6/6	0.97	0.07	0.22	19,22,24,27	0
2	GOL	A	401	6/6	0.97	0.06	-0.15	20,21,24,25	0

6.5 Other polymers (i)

There are no such residues in this entry.

CONFIDENTIAL VALIDATION REPORT
